# Mirabilite with Ice Pack after Total Knee Arthroplasty: A Randomized Controlled Trial Study

**DOI:** 10.1155/2021/6611614

**Published:** 2021-01-12

**Authors:** Ying Zhong, Cheng Zheng, Wenxi Du, Jiahui Zheng, Shanchun Xu, Peijian Tong

**Affiliations:** ^1^Department of Orthopaedics, The First Affiliated Hospital of Zhejiang Chinese Medical University, Hangzhou, Zhejiang 310006, China; ^2^The First Clinical Medical College of Zhejiang Chinese Medical University, Hangzhou, Zhejiang 310053, China; ^3^Department of Orthopaedics, Xianju People's Hospital, Xianju, Taizhou, Zhejiang 317300, China; ^4^Department of General Medicine, The First Affiliated Hospital of Zhejiang Chinese Medical University, Hangzhou, Zhejiang 310006, China

## Abstract

**Background:**

Total knee arthroplasty (TKA) is a well-established procedure for end-stage arthritis of the knee with complications such as swelling and pain. The aim of this study is to estimate the effect of mirabilite with ice pack versus ice pack in relieving pain, swelling, range of motion (ROM), and serum CRP level on patients after TKA.

**Methods:**

Eighty patients undergoing primary unilateral TKA were randomly assigned to two groups (MIP group and WIP group). We used VAS to measure knee pain at 24 h, 48 h, and 72 h after the surgery, respectively. Knee swelling degree was evaluated by measuring the girth of the leg at the center of the patella and 10 cm above and below it at the same frequency. The active ROM of the knee was measured by using a universal goniometer of plastic material at 72 h postoperatively. The serum CRP level was also measured at 72 h postoperatively.

**Results:**

The MIP group showed statistically significant lower knee girth at 48 h (*p* < 0.05) and 72 h (*p* < 0.05) postoperatively and VAS score at 72 h (*p*=0.018) postoperatively after TKA than the WIP group. The ROM of the MIP group was significantly wider than the WIP group (*p*=0.024). The CRP level (*p*=0.036) and length of stay (LOS) (*p*=0.037) of the MIP group were significantly lower than the WIP group.

**Conclusion:**

Mirabilite with ice pack after TKA showed superiority in relieving pain, reducing swelling, and improving ROM than ice pack only.

## 1. Introduction

Total knee arthroplasty (TKA) is a well-established procedure for end-stage arthritis of the knee that has been demonstrated to improve pain, mobility, function, and quality of life [1]. Patients treated with TKA often experience localized swelling and pain resulting from tissue damage and inflammatory response, which usually peaks 24 to 48 hours after surgery and influence postoperative opiate use, a requirement for blood transfusion and postoperative rehabilitation [2]. This can further cause an increased length of stay (LOS) and cost [3].

Nonpharmaceutical treatment such as cryotherapy plays a role in addressing immediate postoperative complications such as pain and swelling [2]. Basic cryotherapy includes basic gel packs and crushed ice. New generation cryotherapy such as electronic temperature control device can guarantee a sustained fixed temperature during cooling to achieve a better effect, but with higher costs [4].

Mirabilite, known as Glauber's salt, is a hydrous sodium sulfate mineral with the chemical formula Na_2_SO_4_·10H_2_O that has the function of reducing inflammation and swelling [5]. In China, ice pack used with mirabilite is considered efficient in reducing swelling and pain after trauma [6, 7]. Mirabilite has a much lower economic cost than new generation cryotherapy, an ideal choice for patients with financial difficulties if proved effective.

The aim of this study is to estimate the effect of mirabilite with ice pack versus ice pack only in relieving pain, swelling, and improving range of motion (ROM) on patients after TKA.

## 2. Materials and Methods

### 2.1. Study Design

This is a single-center, randomized controlled trial (level of evidence: 1).

All patients who underwent single-stage TKA from January 2017 to March 2018 in the department of orthopedics, the First Affiliated Hospital of Zhejiang Traditional Chinese Medicine University, were included.

Inclusion criteria were unilateral primary TKA, severe osteoarthritis (in Kellgren–Lawrence grade 4), and 65–85 years old.

Exclusion criteria were severe varus or valgus deformity, hematological or rheumatologic osteoarthritis etiologies, history of deep vein thrombosis, active phlebitis, venous insufficiency, a coagulation disorder, untreated diabetes, untreated hypertension, and skin damage or sensibility alterations (e.g., hypersensitivity to cold and mirabilite).

The sample size was calculated a priori by G*∗*Power 3.1. Data from a previous study [8] in our center was used to estimate effect size and correlation among repeated measures for a repeated measure ANOVA design; the alpha error probability is 0.05, and power (1-beta) is 0.8. The minimum total sample size is 72.

Eighty patients were included (38 males, 42 females) and randomly assigned to two groups: the water ice pack (WIP) group (*n* = 40) received traditional cryotherapy (crushed ice packs) and the mirabilite with ice pack (MIP) group (*n* = 40) with mirabilite packs under ice bags. In this study, we apply simple randomization using sealed opaque randomization envelopes containing equal numbers of “WIP” and “MIP” notes. There were no dropouts or withdrawals. In the WIP group, an impermeable PVC pack with 500 g of crushed ice packed in was put on the patella and pressurized secured by gauzes. In the MIP group, 500 g of granular mirabilite was spread in a 25 cm*∗*30 cm textile pack and placed around the knee joint before applying the ice pack like the WIP group. In both the two groups, cryotherapy was applied in the first 6–72 h after the operation. The surgeons replaced mirabilite packs and ice packs every 12 h and paid attention to the limb sensory, range of motion, and local skin color.

The visual analog scale (VAS) pain scores at 24, 48, and 72 h after the operation, the difference between the knee girth (the girths at the center of the patella (S0) and 10 cm below (S1) and 10 cm above (S2) the center of patella) at 24, 48, and 72 h after the operation and before the operation, ROM at 72 h postoperatively of the knee, CRP at 72 h postoperatively, and the length of hospital stay were the primary outcomes.

The degree of swelling reflected by knee girths difference at 24, 48, and 72 h after the operation. CRP as an indicator of inflammation was measured 72 hours after the operation.

In addition, the active ROM of the knee was measured using a universal goniometer of plastic material at 72 h after the operation according to Daniel et al. [9]. The goniometer was positioned on the articular line of the knee; the fixed arm was parallel to the lateral surface of the femur in the direction of the greater trochanter and the mobile arm was parallel to the lateral side of the fibula in the direction of the lateral malleolus.

### 2.2. Arthroplasty Procedures

The arthroplasty procedures were carried out by the same group of surgeons, using a midline anterior incision with a medial parapatellar arthrotomy and with the use of posterior cruciate-stabilizing knee prosthesis (Stryker®, NGR) in all patients. The anesthesia and postoperative analgesia protocols used were standardized and similar in the two groups. The mean procedure duration was 70 minutes (60–100). The procedure was performed with a tourniquet in all cases. It was released before closing the wound to realize complete hemostasis. Local injection of 20 ml 5% tranexamic acid into periarticular soft tissue was performed before closing the incision. Patient-controlled analgesia (PCA) pump was applied within 48 h after surgery. Tramadol 100 mg/mL (after 48 hours) was employed for breakthrough pain. Anticoagulant therapy was initiated 6 hours after the end of surgery in all patients. In all patients, the hemorrhage drain was taken out 48 h postoperatively. The same rehabilitation program was used for all patients: partial weight-bearing was allowed, using crutches, since the first day after surgery and progressively increased as tolerated. Active and passive mobilization was started from the first day after surgery. Venous ultrasonography ultrasound was performed when deep vein thrombosis (DVT) was suspected.

### 2.3. Ethical Considerations

This study was approved by the Ethics Committee of the First Affiliated Hospital of Zhejiang Chinese Medical University. The study purpose, methods, and procedures were well explained to all participants at the beginning of this study. All participants signed informed consent and were free to withdraw during the study period.

### 2.4. Statistical Analysis

Median (25–75 percentile) or mean ± standard deviation or percentage was determined for patient baseline demographics, clinical features, and laboratory parameters, when appropriate. Differences between the MIP group and WIP group were evaluated by Student's *t*-test for parametric data, the Mann–Whitney test, or Kruskal–Wallis test for nonparametric data. The chi-square test (*χ*^2^) or Fisher's exact test were used for comparisons of percentages between groups, as appropriate.

Repeated measures ANOVA was performed to evaluate the girth of operative knee and VAS pain score at 3 postoperative time points (24 h, 48 h, and 72 h). Given the ordinal nature of VAS scoring, we used both ANOVA and linear mixed models to compare the treatment effect at different time points. Preliminary tests including detecting extreme outliers, Shapiro–Wilk normality test, and Mauchly's test of sphericity were performed to check if the model assumptions are met. Post hoc tests for significant two-way interaction were decomposed into 2 models: simple main effect (one-way) model, where we evaluate treatment effect at each level of postoperative time points, and simple pairwise comparisons (paired test) to determine which groups are different. The Bonferroni multiple testing correction method was used to adjust *p* values, and compound symmetry correlation structure was applied for linear mixed models. We checked for a correlation matrix between continuous variables by using the Spearman method to compute the correlation coefficient. Statistical analyses were performed using R project version 3.6.0. Package “rstatix” and “nlme” were used for repeated measures ANOVA and linear mixed models. A *p* value <0.05 was considered statistically significant.

## 3. Results

None of the patients experience any skin or vessel complications due to ice pack or mirabilite pack. No infection, DVT, or pulmonary embolism are found after TKA in the 2 groups. The baseline characteristics of the two groups are shown in Table 1. The chi-square test shows no significant difference in gender (*p*=0.258). Also, the age, operation time, BMI, height, weight, postoperative drainage, preoperative VAS score, and preoperative knee girth show no statistically significant difference (*p* > 0.05). The baseline characteristics of the 2 groups are regarded as homogenous. As Table 1 shows, the LOS postoperatively and CRP at 72 h postoperatively in the MIP group are significantly lower than in the WIP group (*p*=0.037) while the ROM at 72 h postoperatively in the MIP group is wider than in the WIP group (*p*=0.024).

The VAS score and girth difference at S0, S1, and S2 have a significant difference in preoperation and postoperation in both the 2 groups. Repeated measure ANOVA shows that the time interaction effect is statistically significant (*F* = 407.9, *p* < 0.001; *F* = 87.3, *p* < 0.001; *F* = 48.6, *p* < 0.001; and *F* = 81.5, *p* < 0.001, resp.). The interaction between treatment and time effect is not statistically significant in VAS score (*p*=0.052). There is statistically significant interaction between treatment and time effect in girth difference at S0, S1, and S2 (*p* < 0.05).

**Figure 1:**
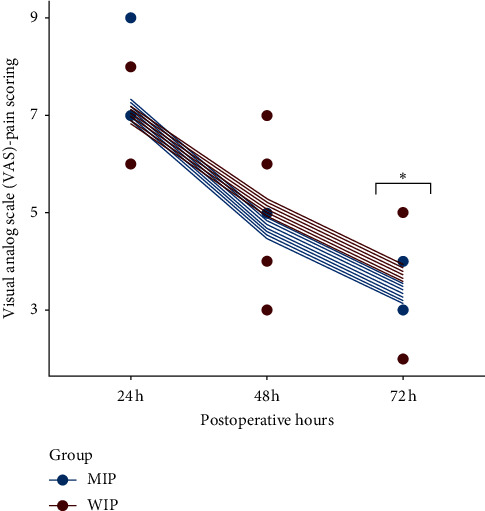
Deviation of the VAS score postoperatively of the 2 groups. The predicted VAS score at the individual level for different postoperative hours. A significant difference is found at 72 h after surgery in a pairwise comparison.

The analysis of anova on post-treatment result shows that VAS scores at 72 h in the MIP group are significantly lower than in the WIP group (3 vs. 4, *p*=0.018).There is no statistically significant difference in VAS score at 24 h and 48 h postoperatively between the 2 groups (*p* > 0.05) (Figure 1).

**Figure 2:**
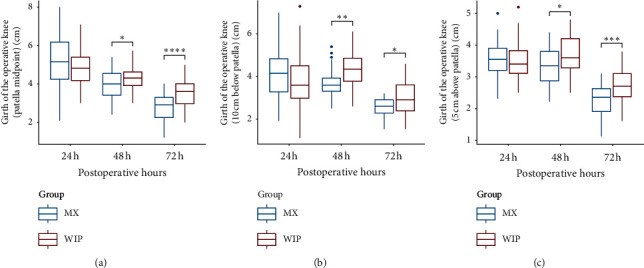
Comparison of knee girth difference between the two groups.

There is no statistically significant difference in knee girth difference on S0, S1, and S2 (*p* > 0.05) at 24 h postoperatively. Girth differences on S0, S1, and S2 at 48 h (*p*=0.045; *p*=0.002; and *p*=0.022, resp.) and 72 h (*p* < 0.001; *p*=0.014; and *p* < 0.001, resp.) postoperatively in the MIP group are significantly lower than in the WIP group (Figure 2).

**Figure 3:**
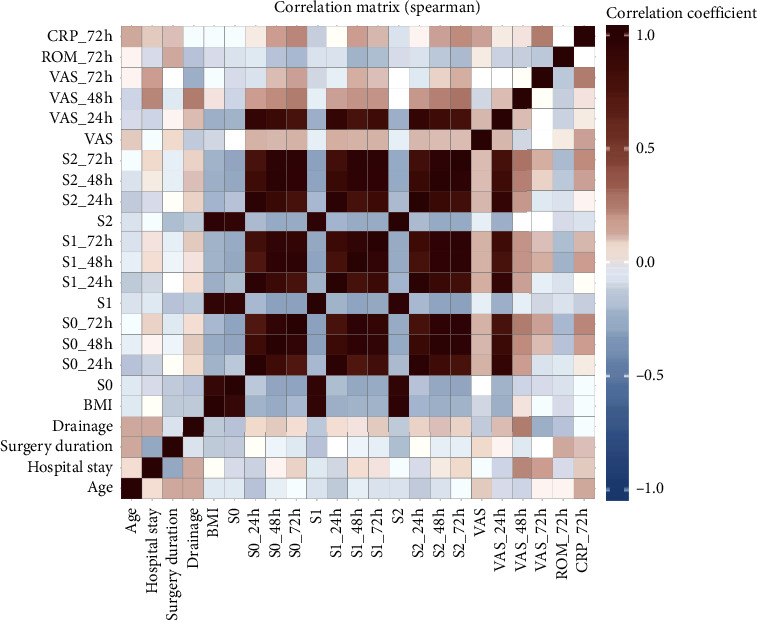
Result of the Spearman test.

The results of the Spearman test are shown in Figure 3. There was no statistically significant correlation between CRP and ROM, VAS, and girth difference at 72 h postoperatively.

## 4. Discussion

Total knee arthroplasty is one of the most cost-effective and consistently successful surgeries performed in orthopedics. Despite the progress in surgical techniques and postoperative care, complications such as swelling and pain become the key of optimizing patients' satisfaction [1].

In our study, the mean girths of the knee joint strikingly increased after surgery in both groups, as was described in previous studies [10]. In conformity with a previous study, the pain score increased to the highest level within 24 h after TKA [11]. This is probably due to the variable intraoperative soft tissue trauma with consecutive secretion, bleeding, and swelling as well as individual postoperative reaction [4]. Blooding and damaged tissue caused by surgery will release inflammatory mediators like TNF-*α*, IL-1*β*, IL-6, and IL-8, increasing vascular permeability, causing inflammatory exudation, and thus leading to swelling and pain which is acute phase response (APR). APR is a systemic reaction to tissue injury and does not vary in a significantly different way in surgical techniques [12].

Cryotherapy is a nonpharmaceutical treatment to the relief of postoperatively pain and swelling, which involves the application of cold to the skin surrounding the injured soft tissues and, in joint surgery, is supposed to reduce the temperature in the knee joint [10]. The low temperature will reduce local blood flow through vasoconstriction, which relieves the local inflammatory reaction, swelling, and heat experience and also will slow the conduction of nerve signals potentially reducing pain transmission [13–15]. In addition, the previous study has shown that a pleasant sensation evoked by knee icing also will reduce the pain intensity after TKA [16]. Kullenberg et al.'s [17] study showed water ice packs were more effective than epidural analgesia in reducing pain degree after TKA. Kuo et al. [18] also confirmed the effect of an ice pack on antiswelling and analgesic. Analysis from Thacoor and Sandiford [19] showed that cryotherapy had certain efficacy in reducing swelling and pain degrees after TKA. Cryotherapy is applied to reduce the degree of swelling and pain whether in theory or clinical practice after trauma [20].

Several cryotherapy options are available including first-generation cold therapy like crushed ice in a plastic pack, new generation advanced computer-assisted devices with continuous controlled cold therapy [2]. The advantage of these latter devices is controlled-temperature modulation with cooling at a specific and continuous temperature for a prolonged time, introducing a better effect of resolving pain and swelling than crushed ice pack [21]. Chughtai et al. [22] reviewed 16 studies where various types of cryotherapy were assessed and compared, concluding that continuous temperature devices are the most effective. However, using the new generation means a much higher cost than traditional cryotherapy, which is unaffordable for most patients.

Mirabilite is a cheap hydrous sodium sulfate mineral which has the special physical properties of salt and is always applied externally to relieve pain and swelling after trauma in traditional Chinese medicine. In traditional Chinese medical theory, mirabilite has a feature of clearing fire-evil which, in modern medical theory, we call inflammation and absorbing swelling. Some Chinese scholars show that by inhibiting the reticuloendothelial system, mirabilite has a positive effect on anti-inflammation. Lu et al.'s [23] study in rabbits shows that mirabilite can significantly decrease the expression of IL-1, IL-6, TNF-*α,* and the severity of inflammatory responses. Wang et al.'s study shows that, for severe acute pancreatitis patients, the group that used hot compresses with mirabilite takes a shorter time for abdominal distension, abdominal pain, and blood and urinary amylase values to normalize and get the lower level of white blood cell count, C-reactive protein, and interleukin-6 level than the group with conventional nursing care and therapy, which may be due to the anti-inflammatory effects of mirabilite [24]. In our study, the MIP group shows statistically significant lower knee girth at 48 h and 72 h postoperatively and VAS score at 72 h postoperatively than the WIP group, which suggests that mirabilite and ice pack can synergetically control swelling and pain. Although there are slight differences in the improvement of ROM between the two groups, it may suggest the better relief of pain and swelling in the MIP group indirectly. In addition, LOS in the MIP group indicates that compared with only ice pack, mirabilite with ice pack can accelerate the recovery process and discharge, therefore reducing unit treatment cost. Inspired by previous studies, we recorded and analysed the postoperative degree of serum CRP. Although there is no statistically significant correlation between CRP and ROM, VAS, and girth difference at 72 h postoperatively, our study shows that serum CRP at 72 h after surgery of the MIP group is significantly lower than the WIP group. These results support the viewpoints described above. The anti-inflammatory function of mirabilite may be the reason for the better postoperative performance in the MIP group. However, the mechanism of mirabilite anti-inflammatory and reducing pain and swelling still needs to be further confirmed and studied.

In addition, mirabilite as an osmotic laxative and a stimulant laxative have an intense effect on contracting the intestinal smooth muscle and increasing peristalsis. Zhong et al.'s study confirms that after stimulating the large intestine by mirabilite, SP, NK1R, VIP, VIPR1, and VIPR2 were all significantly increased in the large intestine tissue of rats [25]. However, in our study, there was no intestinal reaction and other side-effects of other systems had been found in the MIP group. Although some previous studies reported on frostbite, deep vein thrombosis, and further adverse effects after cryotherapy [17], there was no occurrence of any adverse event reported in the 2 groups throughout our study. Our study shows that the use of cryotherapy and mirabilite after TKA is safe.

## 5. Conclusions

Our study shows that mirabilite with ice pack in patients undergoing TKA is significantly more effective than ice pack with regard to reducing swelling, pain, CRP level, LOS, and improving ROM, which maybe due to anti-inflammatory property of the mirabilite. Appling mirabilite with ice pack externally after TKA appears to be a cheap, safe, and efficient procedure in clinical practice.

## Figures and Tables

**Table 1:** Comparison of baseline characteristics and clinical features between the 2 groups.

		MIP group	WIP group	*p* value
Male		20 (50.00%)	14 (35.00%)	0.258
Age (years)		72.00 (69.00–76.00)	70.50 (69.00–73.00)	0.275
Weight (kg)		69.40 (64.67–74.48)	69.10 (63.15–75.55)	0.927
Height (m)		1.64 (1.59–1.71)	1.65 (1.61–1.72)	0.443
BMI (kg/m^2^)		25.56 (22.90–27.67)	25.17 (23.40–27.29)	0.658
Operation time (min)		68.00 (64.00–75.25)	70.00 (65.75–77.25)	0.383
Postoperative drainage (mL)		41.50 (33.75–48.00)	41.00 (35.00–47.50)	0.912
Preoperative VAS score		5.00 (4.00–6.00)	5.00 (4.00–5.00)	0.806
Preoperative girth (cm)	S0	40.00 (38.68–42.90)	41.85 (39.77–45.12)	0.077
	S1	34.50 (32.90–37.23)	36.50 (33.20–39.40)	0.179
	S2	45.40 (42.58–48.52)	45.50 (42.85–49.62)	0.577
Length of hospital stay (LOS) (days)		9.00 (8.00–10.00)	8.00 (7.00–9.25)	**0.037**
ROM at 72 h postoperatively		72.00 (67.75–75.00)	74.50 (70.00–80.00)	**0.024**
CRP at 72 h postoperatively (mg/dL)		20.50 (18.00–27.00)	18.00 (14.75–22.00)	**0.036**

BMI, body mass index. Boldface indicates *p* value <0.05.

## Data Availability

The datasets used and analysed during the current study are available from the corresponding author on reasonable request.

## Abbreviations

TKA: Total knee arthroplasty
ROM: Range of motion
CRP: C-reactive protein
VAS: Visual analog scale
BMI: Body mass index
LOS: Length of stay
WIP: Water ice pack
MIP: Mirabilite with ice pack
PVC: Polyvinyl chloride
PCA: Patient-controlled analgesia
DVT: Deep vein thrombosis
ANOVA: Analysis of variance
APR: Acute phase response
TNF: Tumor necrosis factor
IL: Interleukin.
